# Urtica Moroides, or Laportea Moroides

**Published:** 1895-02

**Authors:** Alfred Heath


### URTICA MOROIDES, OR LAPORTEA MOROIDES.

ALFRED HEATH, M. D., F. L. S., ETC.

This virulent plant belongs to Urticaceæ. I adhere to the old name of *Urtica:* 1st. Because it belongs to the same genus as the plant known in homœopathic medicine as *Urtica-gigas* (now called *Laportea-gigas*), the stinging tree of Australia, growing at times to one hundred and forty feet high and twenty feet in circumference. 2d. Because it was originally named *Urtica,* and also that we have in use two or three other *Urticas,* and, as we are familiar with the name of Urtica, and they are all closely related, it is better to group them for the sake of analogy. The slightest touch of *Urtica Moroides* (called Moroides on account of its mulberry-like fruit; which also, although tempting to the eye, is very poisonous, and irritating to the touch) produces the most severe symptoms, and I should very much pity any one who got badly stung by it.

On the 4th of May, 1894, at mid-day, I touched one of my fingers with a single bristle of the living plant growing in the Royal Botanic Gardens, under glass. In a moment there was a red spot with a white blister, followed by sharp, pricking pains like needles, with burning soreness, which lasted very severely all day. The pain was aggravated by warm water, and extremely so by cold water. I felt nothing of it in the night. The whole of the following day, the 5th, it felt bruised and sore, with great itching. At intervals it quite subsided, only to recur, and if, when free from pain, I put cold water on it, or even moistened the spot with my tongue, the sore, burning, itching, pricking came on again, severely for a few minutes. On the 6th it felt bruised, stiff, and itched very much at times, and was relieved by rubbing. May 8th, feeling at times as of subcutaneous soreness; 9th, occasional pricking in the part stung; 19th, accidentally *pressed* on the part stung—felt great subcutaneous soreness, as if pressing on a wound; 30th, same as on the 19th, and for some weeks longer I had occasional reminders, especially on washing my hands in cold water.

The antidote to the painful symptoms produced by this plant is

TINOSPORA CORDIFOLIA.

I have since stung my fingers with the *Urtica Moroides*, and, by applying the juice of Tinospora, which is very abundant in the plant, the pain is at once removed, although it occurs slightly at times. *Tinospora Cordifolia* belongs to the order *Meorispermaceæ*, to which also our well-known *Cocculus Indicus* (*Anamirta Cocculus*) belongs. It probably contains the same alkaloid (*Picrotoxin*) as is found in *Cocculus Indeas*, which we all know produces giddiness, vomiting, convulsions, and insensibility, and, in the small dose, is so good in many forms of sickness. In the proving of the mother tincture of Tinospora, recorded in HOMŒOPATHIC PHYSICIAN of November last by P. Banerger, it produced nausea and vomiting. Tinospora Cordifolia contains a bitter principle, *Columbine*, common to plants of this genus, many of which have tonic and emetic properties, like Nux-vomica. An extract called *Galuncha* is prepared from it. It is considered specific for the bites of poisonous insects, and snake-bites, and for ulcers, and it is, as I have found, specific for the stings of *Urtica*. The young shoots are used as emetics. A curious feature of this plant is its extreme *vitality*. When the main stem is cut or broken in two, a root is sent down from *above*, and continues to grow until it reaches the ground, and thus restores communication. Its wonderful vitality may be some indication for its use in disease, especially to some of our friends who observe the workings of the "doctrine of signatures."

